# Immediate Glycemic Outcomes Following Simultaneous Pancreas–Kidney Transplantation: Equivalent Early Metabolic Profiles in Type 1 and Type 2 Diabetes

**DOI:** 10.7759/cureus.102422

**Published:** 2026-01-27

**Authors:** Mojgan Jalalzadeh, Zahidul Mondal, Nooruddin Hashmi, Sonalis Bunin, Mital Shah, Jing Shen, Daniel P Pieloch, Steve Khalil, Neeraj Singh, Advaith Bongu, Ronald P Pelletier

**Affiliations:** 1 Internal Medicine/Nephrology/Transplant, Rutgers Robert Wood Johnson Medical School, New Brunswick, USA; 2 Internal Medicine-Nephrology, Rutgers Robert Wood Johnson Medical School, New Brunswick, USA; 3 Internal Medicine/Nephrology/ Transplant, Rutgers Robert Wood Johnson Medical School, New Brunswick, USA; 4 Internal Medicine/Nephrology/Transplant, RWJBarnabas Health, New Brunswick, USA; 5 Research, Medicine, Division of Gastroenterology, Rutgers Robert Wood Johnson Medical School, New Brunswick, USA; 6 Kidney, Pancreas and Heart Transplant, RWJBarnabas Health, New Brunswick, USA; 7 Internal Medicine/Nephrology, Rutgers Robert Wood Johnson Medical School, New Brunswick, USA; 8 Surgery, Kidney and Pancreas Transplant, RWJBarnabas Health, New Brunswick, USA; 9 Surgery/Transplant, RWJBarnabas Health, New Brunswick, USA

**Keywords:** delayed graft function, glycemic control, insulin independence, postoperative outcomes, simultaneous pancreas-kidney transplantation, type 1 diabetes mellitus, type 2 diabetes mellitus

## Abstract

Background and objective: Simultaneous pancreas-kidney transplantation (SPKT) is increasingly performed in both type 1 and carefully selected type 2 diabetes mellitus recipients. Although long-term outcomes are well described, early postoperative glycemic behavior remains poorly defined. Persistent hyperglycemia during the early postoperative phase raises concerns regarding potential technical complications or acute rejection after SPKT. In contrast, early normalization of glucose levels suggests pancreas graft viability and sufficient functional beta-cell mass. Despite extensive long-term data, comparative evidence between T1DM and T2DM recipients is limited. This study compares early glycemic trajectories, perioperative glycemic management, and short-term outcomes between these groups.

Methods: We conducted a single-center retrospective study of adult SPKT recipients from January 2018 to December 2025. Exclusion criteria were multiorgan transplantation beyond SPKT, pancreas graft loss or patient death within 24 hours, and incomplete early postoperative glucose data. Perioperative glucose interventions, pancreatic enzyme markers, and glucose values obtained at predefined postoperative time points (6, 12, and 24 hours; POD 7, 14, and 28) were analyzed. Primary outcomes included early glucose levels and insulin independence at one month, a clinically meaningful indicator of endocrine graft function. Secondary outcomes included delayed graft function, postoperative complications, return to the operating room, pancreatic enzyme levels, and length of hospital stay.

Results: Among 80 SPKT recipients, 31 (38.8%) had T1DM and 49 (61.2%) had T2DM. T2DM recipients were older (50.3 ± 9.3 vs. 41.3 ± 9.3 years, p = 0.001) and had a higher body mass index (27.0 ± 3.5 vs. 24.9 ± 4.0 kg/m², p = 0.016). T1DM recipients, in contrast, had a longer duration of diabetes and lower C-peptide levels (both *p* < 0.001). Early postoperative glucose values at 6, 12, and 24 hours, as well as on postoperative days 7, 14, and 28, were similar between groups (all *p* > 0.05). Rates of intravenous insulin use (T1DM: 3/31 [9.7%] vs. T2DM: 7/49 [14.3%]; p = 0.732) and intravenous dextrose administration (23/31 [74.2%] vs. 26/49 [53.1%], p = 0.191) were comparable. No significant differences were observed in pancreatic enzyme levels, insulin dependence at one month, delayed graft function, postoperative complications, or length of hospital stay.

Conclusions: Early postoperative glycemic control and short-term graft outcomes are comparable between type 1 diabetes mellitus (T1DM) and type 2 diabetes mellitus (T2DM) SPKT recipients, supporting the safety and applicability of standardized perioperative glycemic protocols across both populations.

## Introduction

Simultaneous pancreas-kidney transplantation (SPKT) is a well-established treatment for patients with insulin-dependent diabetes mellitus and end-stage renal disease, providing excellent patient and graft survival in the modern era [1-3]. Historically, SPKT was predominantly restricted to individuals with type 1 diabetes mellitus (T1DM). However, advances in understanding diabetes heterogeneity, improvements in perioperative management, and refined recipient selection criteria have expanded its application to carefully selected patients with type 2 diabetes mellitus (T2DM) [4-8].

Multiple registry-based and single-center studies have demonstrated that T1DM recipients and appropriately selected T2DM recipients can achieve comparable long-term outcomes after SPKT or pancreas transplantation alone [4-8]. Although metabolic normalization following pancreas transplantation is often rapid, the early postoperative period is particularly critical, as glycemic trends may reflect graft function, surgical complications, or early immunologic injury [9-13]. Studies using continuous glucose monitoring (CGM) have shown that, despite predominant normoglycemia, early episodes of hyperglycemia, hypoglycemia, and glycemic variability are not uncommon and may carry prognostic significance [10-13]. Early postoperative glycemic patterns offer valuable, real-time insights into pancreatic graft function and potential perioperative complications. These immediate readings can be more clinically informative than long-term glycemic outcomes, which may be affected by various factors over time, including immunologic responses, metabolic changes, and recipient characteristics.

Early hyperglycemia following the transplant can increase stress on beta cells and worsen ischemia-reperfusion injury, potentially making recipients more susceptible to issues such as pancreatitis, vascular thrombosis, or early graft dysfunction. In contrast, effective early glycemic control may provide protective benefits to the pancreas allograft. Additionally, maintaining tight glycemic control has been linked to reduced wound complications and infection risk, which are particularly common among patients with SPKT [14-16].

Despite these observations, early postoperative glycemic patterns remain incompletely characterized, especially in direct comparisons between T1DM and T2DM SPKT recipients. Accordingly, this study aimed to describe early glycemic trajectories in T1DM and T2DM recipients following SPKT and to evaluate associated short-term outcomes, including delayed graft function (DGF), postoperative complications, and length of hospital stay.

The objective of this study was to compare early postoperative glycemic trajectories and short-term metabolic outcomes between type 1 and type 2 diabetes mellitus recipients of SPKT in order to evaluate whether diabetes phenotype influences early graft function under standardized perioperative care.

## Materials and methods

Study design and setting

This retrospective study included adult recipients of simultaneous pancreas-kidney transplantation (SPKT) at a single academic medical center between January 2018 and December 2025. The study was approved by the institutional review board (IRB Pro2025002041), and informed consent was waived.

Eligible participants were adults undergoing primary SPKT during the study period. Exclusion criteria included multiorgan transplantation beyond SPKT, pancreas graft loss or patient death within 24 hours of transplantation, and incomplete early postoperative glucose data. The type of diabetes was determined by transplant endocrinology based on clinical phenotype, medical history, and objective metabolic parameters in alignment with OPTN/UNOS candidate reporting criteria for kidney-pancreas transplantation, including insulin dependence, C-peptide levels, and body mass index (BMI). All patients received standardized induction immunosuppression with rabbit anti-thymocyte globulin (thymoglobulin) and methylprednisolone. Maintenance immunosuppression consisted of prednisone, an antimetabolite agent, and a calcineurin inhibitor.

Data collection and glucose monitoring protocols

Recipient variables included age, sex, race/ethnicity, BMI, duration of diabetes, C-peptide level, calculated panel reactive antibody (cPRA), and human leukocyte antigen (HLA) mismatch. Donor characteristics included age, sex, race/ethnicity, BMI, donor type (donation after brain death [DBD] versus donation after circulatory death [DCD]), terminal amylase and lipase levels, kidney cold ischemia time, and pancreas preservation time.

Perioperative glycemic management included an intravenous insulin infusion and dextrose 10% (D10) administration during the first 24 hours following transplantation. Glucose monitoring was performed using point-of-care capillary measurements at 6, 12, and 24 hours postoperatively, and again on postoperative days (POD) 7, 14, and 28. In addition to these predefined time points, glucose was monitored every 4 hours during the first 24 hours and every 8-12 hours thereafter until POD 7, per institutional protocol, to ensure safety and to capture clinically significant excursions.

Insulin infusion targets were a blood glucose range of 120-180 mg/dL, with adjustments made per standardized insulin titration algorithms. Insulin infusion was initiated for glucose >180 mg/dL and titrated per protocol to avoid both hyperglycemia and hypoglycemia. Dextrose 10% (D10) was administered when glucose levels fell below 100 mg/dL or when hypoglycemic symptoms occurred, according to preset thresholds in the postoperative glycemic management protocol.

Early pancreas graft markers included peak amylase and lipase levels within the first seven postoperative days, as well as values measured at day 30.

Handling of missing data and protocol deviations

Missing glucose or laboratory values at select time points were handled using pairwise deletion, and sensitivity analyses were performed to ensure robustness to missingness; missing data were limited and evenly distributed across T1DM and T2DM groups. There were no significant protocol deviations during the study period, and all patients received the same standardized perioperative care, except when clinically contraindicated.

Outcomes

Primary early outcomes included mean blood glucose levels during the first 24 hours after SPKT, glycemic values at all measured time points, and insulin dependence at one month. Secondary outcomes included delayed graft function (DGF), defined as the need for dialysis within the first postoperative week; 30-day surgical complications; return to the operating room within 30 days; and length of hospital stay.

Statistical analysis

Continuous variables were reported as mean ± standard deviation (SD) or median [interquartile range, IQR], as appropriate. Categorical variables were reported as counts (n) and percentages (%), with percentages calculated from the total number of evaluable subjects in each group. Group comparisons were performed using Student’s t-test for normally distributed continuous variables, the Mann-Whitney U test for non-normally distributed continuous variables, and chi-square or Fisher’s exact test for categorical variables, as appropriate. Linear regression models were used to fit the association between post-transplant time and blood glucose levels in both T1DM and T2DM groups and estimate the regression equation, coefficient of determination (R2), and corresponding p-value for each group. All tests were two-sided, with statistical significance defined as p < 0.05.

T2DM recipients were analyzed as a single group because all candidates met uniform, predefined SPKT selection criteria, including insulin dependence, preserved but inadequate endogenous insulin production, and OPTN/UNOS metabolic thresholds, which minimize metabolic variability relevant to early postoperative glycemic behavior and support group-level analysis.

## Results

Baseline characteristics

A total of 80 SPKT recipients met the inclusion criteria, including 31 (38.8%) with type 1 diabetes mellitus (T1DM) and 49 (61.2%) with type 2 diabetes mellitus (T2DM). Recipient baseline characteristics are summarized in Table 1. T2DM recipients were significantly older than T1DM recipients (50.3 ± 9.3 vs. 41.3 ± 9.3 years; t = 4.23, p = 0.001) and had a higher body mass index (27.0 ± 3.53 vs. 24.9 ± 3.98 kg/m²; t = 2.47, p = 0.016). T1DM recipients had a longer duration of diabetes (median 24.0 [IQR 18.5-34.0] vs. 18.5 [14.8-23.0] years; U = 1009.5, p < 0.001) and significantly lower C-peptide levels (0.005 [0, 1.32] vs. 6.94 [3.1, 10.0] ng/mL; U = 84, p < 0.0001).

**Table 1:** Baseline donor and recipient characteristics Data are presented as mean ± SD, median [IQR], or n (%). Percentages were calculated using evaluable subjects in each group. Comparisons used Student’s t-test, Mann–Whitney U test, chi-square test, or Fisher’s exact test, as appropriate. Statistical significance was defined as p < 0.05.

Variable	T1DM (n = 31)	T2DM (n = 49)	Test statistics	p-value
Recipient Characteristics				
Age, years (mean ± SD)	41.3 ± 9.26	50.3 ± 9.30	t=4.23	0.001
Sex, n (%)				
Female	5 (16.1%)	11 (22.4%)	c^2^=0.47	0.491
Male	26 (83.9%)	38 (77.6%)		
Race/Ethnicity, n (%)				
Asian, non-Hispanic	3 (9.7%)	14 (28.6%)	c^2^=7.38	0.061
Black, non-Hispanic	8 (25.8%)	11 (22.4%)		
Hispanic/Latino	8 (25.8%)	16 (32.7%)		
White, non-Hispanic	12 (38.7%)	8 (16.3%)		
BMI (kg/m²), mean ± SD	24.9 ± 3.98	27.0 ± 3.53	t=2.47	0.016
Diabetes duration, years (median [IQR])	24.0 [18.5, 34.0]	18.5 [14.8, 23.0]	U=1009.5	<0.001
C-peptide (ng/mL), median [IQR])	0.005 [0, 1.32]	6.94 [3.1, 10.0]	U=84	<0.0001
cPRA (%), mean ± SD	13.8 ± 27.5	11.7 ± 21.0	t=0.34	0.737
HLA mismatch (count), mean ± SD	4.65 ± 1.23	4.92 ± 1.02	t=1.04	0.305
Donor Characteristics				
Donor age, years (mean ± SD)	31.7 ± 10.2	29.8 ± 11.9	t=0.76	0.453
Donor sex, n (%)				
Female	18 (58.1%)	17 (34.7%)	c^2^=3.32	0.069
Male	13 (41.9%)	32 (65.3%)		
Donor Race/Ethnicity, n (%)				
Asian, non-Hispanic	0 (0%)	4 (8.2%)	Fisher’s exact test	0.022
Black, non-Hispanic	2 (6.5%)	13 (26.5%)		
Hispanic/Latino	6 (19.4%)	8 (16.3%)		
Multiracial	0 (0%)	2 (4.1%)		
White, non-Hispanic	22 (71.0%)	22 (44.9%)		
Donor BMI (kg/m²), mean ± SD	21.2 ± 10.1	22.9 ± 11.2	t=0.70	0.489
Donor type: DCD, n (%)	6 (19.4%)	7 (14.3%)	c^2^=0.08	0.774
Donor type: DBD, n (%)	27 (87.1%)	43 (87.8%)	c^2^=0	1.00
Donor terminal amylase (U/L), mean ± SD	104 ± 143	97.1 ± 129	t=0.22	0.827
Donor terminal lipase (U/L), mean ± SD	38.5 ± 41.7	42.6 ± 44.9	t=0.40	0.694
Kidney cold ischemia time (hours), mean ± SD	14.6 ± 3.15	13.5 ± 2.39	t=1.73	0.089
Pancreas preservation time (hours), mean ± SD	12.5 ± 2.59	11.5 ± 2.25	t=1.75	0.085
KDPI (%), mean ± SD	17.2 ± 10.6	17.9 ± 15.0	t=0.26	0.797

There were no significant differences between groups in calculated panel reactive antibody (cPRA), degree of HLA mismatch, or recipient sex distribution. Recipient race/ethnicity distributions differed numerically but did not reach statistical significance (χ² = 7.38, p = 0.061).

Donor characteristics were broadly comparable between groups, including donor age, donor body mass index, donor type (donation after circulatory death vs. donation after brain death), kidney cold ischemia time, pancreas preservation time, and Kidney Donor Profile Index (KDPI). Donor race/ethnicity distribution differed significantly between groups (Fisher’s exact test, p = 0.022), while donor sex distribution showed a nonsignificant trend (χ² = 3.32, p = 0.069). Terminal donor amylase and lipase levels were similar between groups.

Perioperative glycemic management and early graft markers

Perioperative glycemic management and early graft markers are summarized in Table 2. The proportion of recipients requiring intravenous insulin infusion during the first 24 postoperative hours did not differ between groups (T1DM: 3/31 [9.7%] vs. T2DM: 7/49 [14.3%]; Fisher’s exact test, p = 0.732). Intravenous dextrose (D10) administration was also comparable between T1DM and T2DM recipients (23/31 [74.2%] vs. 26/49 [53.1%]; χ² = 1.68, p = 0.191).

**Table 2:** Perioperative glycemic management and early graft markers Continuous variables are presented as mean ± SD, and categorical variables are reported as n (%). Comparisons used Student’s t-test, Mann–Whitney U test, chi-square test, or Fisher’s exact test, as appropriate. A p-value < 0.05 was considered statistically significant.

Variable	T1DM (n=31)	T2DM (n=49)	Test statistics	p-value
IV insulin infusion, n (%)	3 (9.7%)	7 (14.3%)	Fisher’s exact test	0.732
IV dextrose (D10) given, n (%)	23 (74.2%)	26 (53.1%)	c^2^=1.68	0.191
Peak amylase (U/L, first 7 days)	94.3 ± 55.1	130.1 ± 106	t=1.62	0.109
Peak lipase (U/L, first 7 days)	75.8 ± 87.9	99.2 ± 108	t=0.99	0.325
Day-30 amylase (U/L)	89.0 ± 22.7	101.1 ± 43.7	t=1.18	0.241
Day-30 lipase (U/L)	65.4 ± 58.5	59.0 ± 43.5	t=0.538	0.592

Peak serum amylase and lipase levels within the first seven postoperative days did not differ significantly between groups. Similarly, serum amylase and lipase levels on postoperative day 30 were comparable between recipients with T1DM and those with T2DM.

Early glycemic values

**Figure 1:**
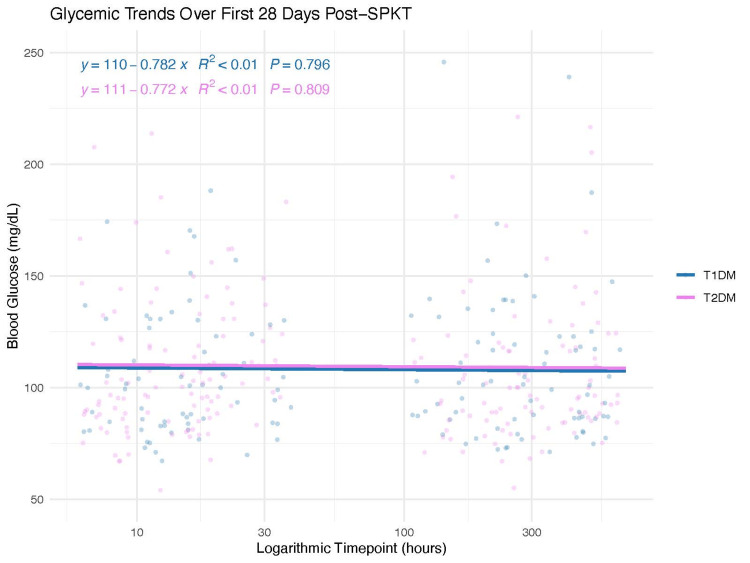
Postoperative glycemic trajectories The scatter plot displayed longitudinal blood glucose measurements collected during the first 28 days post-transplant in patients with T1DM (blue) and T2DM (magenta) who underwent SPKT. Time points have been uniformly converted to hours post-transplant and displayed on a logarithmic scale to better represent sampling data from both early and late post-transplant periods. Individual measurements were displayed as jittered points to reduce overlap. Linear regression lines were fitted separately for T1DM and T2DM using ordinary least squares regression without displaying confidence intervals. Polynomial regression annotations showed the fitted regression equation, coefficient of determination (R^2^), and corresponding p-value for each group. Linear regression analysis revealed no significant association between post-transplant time and blood glucose levels in either group (p > 0.79). The regression slopes for both groups were close to zero, suggesting no significant systematic changes in blood glucose levels during the early and late post-transplant periods.

Mean glucose levels measured at early (6, 12, and 24 hours) and later time points (postoperative days 7, 14, and 28) showed comparable outcomes between recipients with T1DM and those with T2DM (all p > 0.05). Rates of insulin dependence at one month were also comparable between groups (T1DM: 3/31 [9.7%] vs. T2DM: 7/49 [14.3%]; Fisher’s exact test, p = 0.732). These results are visually represented in Figure 1, which depicts the overlapping postoperative glycemic trajectories during the initial 28 days, indicating no significant divergence between the two groups (Table 3).

**Table 3:** Early postoperative glycemic metrics Glucose values are shown as mean ± SD. Insulin dependence is reported as n (%). Between-group comparisons used Student’s t-test or chi-square test, as appropriate. Statistical significance was defined as p < 0.05.

Timepoint	T1DM (n=31)	T2DM (n=49)	Test statistics	p-value
6 hours, mean ± SD	113 ± 29.3	109 ± 41.2	t=0.49	0.626
12 hours, mean ± SD	99.6 ± 27.1	107 ± 34.6	t=1.02	0.310
24 hours, mean ± SD	110 ± 27.3	113 ± 24.8	t=0.44	0.663
POD7, mean ± SD	114 ± 36.8	114 ± 48.5	t=0.006	0.996
POD14, mean ± SD	108 ± 33.2	107 ± 42.9	t=0.07	0.942
POD28, mean ± SD	103 ± 26.7	106 ± 31.7	t=0.36	0.721
Insulin dependence at 1 month, n (%)	3 (9.7%)	7 (14.3%)	Fisher’s exact test	0.732

Early surgical outcomes

Early postoperative surgical outcomes are summarized in Table 4. Rates of delayed graft function were similar between T1DM and T2DM recipients (8/31 [25.8%] vs. 13/49 [26.5%]; Fisher’s exact test, p = 1.00). Use of bladder drainage did not differ between groups (21 [67.7%] vs. 30 [61.2%]; χ² = 0.12, p = 0.725). There were no significant differences in 30-day postoperative complications, including bladder leak, ureteral leak, enteric leak, pancreatic leak, abdominal infection, or wound infection (all p > 0.05). Rates of return to the operating room within 30 days were numerically higher among T2DM recipients but did not reach statistical significance (20/49 [40.8%] vs. 7/31 [ 22.6%]; χ² = 2.07, p = 0.151). Length of hospital stay was similar between groups (median 9.0 [IQR 6.0-10.0] vs. 9.0 [6.5-13.5] days; U = 638.5, p = 0.358).

**Table 4:** Early postoperative surgical outcomes Outcomes are reported as n (%), except length of stay, shown as median [IQR]. Comparisons used the chi-square test, Fisher’s exact test, or Mann–Whitney U test, as appropriate. A p-value < 0.05 was considered statistically significant.

Outcome	T1DM (n=31)	T2DM (n=49)	Test statistics	p-value
Delayed graft function, n (%)	8 (25.8%)	13 (26.5%)	Fisher’s exact test	1.00
Bladder drainage used, n (%)	21 (67.7%)	30 (61.2%)	c^2^=0.12	0.725
30-day complications, n (%)				
Bladder leak	4 (12.9%)	5 (10.2%)	Fisher’s exact test	0.729
Ureter leak	2 (6.5%)	2 (4.1%)	Fisher’s exact test	0.643
Enteric leak	0 (0%)	2 (4.1%)	Fisher’s exact test	0.519
Pancreas leak	3 (9.7%)	3 (6.1%)	Fisher’s exact test	0.672
Abdominal infection	4 (12.9%)	9 (18.4%)	Fisher’s exact test	0.757
Wound infection	3 (9.7%)	5 (10.2%)	Fisher’s exact test	1.00
Return to OR ≤ 30 days, n (%)	7 (22.6%)	20 (40.8%)	c^2^=2.07	0.151
Length of stay, days (median [IQR])	9.0 [6.0, 10.0]	9.0 [6.5, 13.5]	U=638.5	0.358

Indications for reoperation or readmission events are summarized in Table 5. The distribution of event categories, including urinary tract complications, bleeding or hematoma, pancreatic or duodenal complications, intra-abdominal infection, gastrointestinal symptoms, cardiopulmonary or sepsis-related events, and other causes, did not differ significantly between T1DM and T2DM groups (all p > 0.05).

**Table 5:** Indications for reoperation or readmission Data are presented as n (%) of events. Percentages reflect proportions of total events. Comparisons used chi-square or Fisher’s exact test. Statistical significance was defined as p < 0.05.

Category	T1DM Events (n=20)	T2DM Events (n=40)	Test statistics	p-value
Urinary tract complications (leak, UTI, urethritis)	4 (25.0%)	7 (17.5%)	Fisher’s exact test	0.808
Bleeding/hematoma	1 (5.0%)	6 (15.0%)		
Pancreatic or duodenal complications	5 (25.0%)	9 (22.5%)		
Intra-abdominal abscess/infection	7 (35.0%)	12 (30.0%)		
Gastrointestinal symptoms (C. diff, diarrhea)	1 (5.0%)	1 (2.5%)		
Cardiopulmonary / sepsis-related	1 (5.0%)	2 (5.0%)		
Other (fascial issues, Page kidney, miscellaneous)	0 (0%)	3 (7.5%)		

## Discussion

This single-center retrospective study reveals that early postoperative glycemic trajectories after simultaneous pancreas-kidney transplantation (SPKT) are comparable between recipients with type 1 diabetes mellitus (T1DM) and carefully selected recipients with type 2 diabetes mellitus (T2DM). Despite notable differences in baseline metabolism, early glucose measurements taken at standardized postoperative intervals showed no significant differences between the groups. In both T1DM and T2DM recipients, early postoperative glucose patterns aligned with expected stress-related glycemic responses following major surgery and immunosuppression. The later normalization of glucose levels likely indicates recovery of functional endocrine graft activity.

Early postoperative glucose behavior not only reflects the immediate secretory capacity of the transplanted pancreas but also acts as a real-time, clinically accessible indicator of graft viability. In this study, the lack of significant differences in early glycemic trajectories between T1DM and carefully selected T2DM recipients, despite baseline metabolic differences, suggests that initial endocrine graft performance is driven more by graft health and standardized perioperative physiology than by recipient diabetes subtype. This finding has important implications, as indications for SPKT extend to include select T2DM candidates, affirming that early metabolic responses can be interpreted similarly across phenotypes. Moreover, vigilant monitoring of early glucose dynamics, whether through point-of-care testing or continuous glucose monitoring, may provide clinicians with timely information about graft performance and potential complications. Such monitoring facilitates prompt clinical decision-making and supports using early glucose trends as a meaningful clinical endpoint, rather than relying solely on long-term outcomes or diabetes classification.

Additionally, perioperative glycemic management strategies, including intravenous insulin and dextrose administration, as well as short-term outcomes such as insulin independence at one-month, delayed graft function (DGF), postoperative complications, and length of hospital stay, were similar across both diabetes phenotypes.

These findings align with previous registry-based and single-center studies, which indicate that, when appropriate selection criteria are employed, recipients with T2DM can achieve pancreas and kidney graft outcomes similar to those of recipients with T1DM [1,4-8]. This study builds on the existing literature by specifically examining early postoperative glycemic trajectories, a crucial yet underexplored period during which glucose trends may reflect graft viability, potential technical complications, or signs of early immunologic injury.

Previous research has established links between early postoperative hyperglycemia, glycemic variability, and adverse outcomes, including infection, rejection, and graft dysfunction [9-13]. Additionally, analyses utilizing continuous glucose monitoring (CGM) have underscored the potential of early glycemic fluctuations as prognostic indicators [10-12]. However, our findings reveal that early point-of-care measurements are comparable across various diabetes phenotypes. This suggests that the prognostic implications of early glycemic levels may be more relevant within individual recipients rather than being predominantly influenced by diabetes subtypes.

Markers indicative of early pancreatic graft function, such as peak pancreatic enzyme levels observed in the first postoperative week and the amylase and lipase measurements taken on day 30, were comparable across both groups. These findings align with previous literature, as the rates of insulin independence at one month showed no significant differences. This reinforces existing evidence that select recipients with T2DM can achieve endocrine graft outcomes comparable to those of recipients with T1DM following SPKT [4-8].

Early surgical outcomes were consistent and promising, showing no significant differences in rates of delayed graft function, anastomotic complications, infections, or the necessity for reoperation within 30 days between the groups. Although serum creatinine levels were modestly elevated in T1DM recipients at one-month post-transplant (1.9 ± 0.73 vs. 1.5 ± 0.63 mg/dL, p = 0.011), the clinical relevance of this difference remains uncertain. Several factors may contribute to this observation, including variations in recipient age, duration of diabetes, dialysis history, donor-recipient size matching, and early hemodynamic factors not fully addressed in this analysis. It is essential to regard this finding as hypothesis-generating rather than a reflection of inferior kidney graft performance, emphasizing the need for further investigation in larger, multicenter studies with extended follow-up periods.

Clinical implications

The findings of this study hold significant clinical relevance. First, they underscore the metabolic safety and feasibility of SPKT in carefully selected recipients with T2DM, thereby supporting the continued expansion of eligibility criteria provided that proper selection standards are upheld. Second, the observed similarities in early postoperative glycemic behavior across various diabetes phenotypes indicate that standardized perioperative glycemic management protocols can be applied without needing specific modifications based on diabetes type. Lastly, early postoperative glycemic monitoring, whether through point-of-care testing or continuous glucose monitoring (CGM), may prove invaluable for detecting early graft dysfunction or complications, rather than relying on diabetes subtype for outcome stratification.

Limitations

This study is not without its limitations. Its retrospective, single-center design and relatively small sample size may have limited statistical power to detect subtle differences between groups and may restrict generalizability. Glucose measurements were obtained at predetermined postoperative time points using point-of-care testing rather than continuous glucose monitoring, which could underestimate glycemic variability and miss transient excursions that might be clinically meaningful. Additionally, exclusion of select missing glucose and laboratory values could introduce bias, although such missing data were limited and evenly distributed between groups. Comprehensive measures of insulin resistance and detailed pretransplant metabolic profiling were inconsistently available, potentially obscuring nuanced metabolic differences. While minor inconsistencies in table labeling were identified and corrected during manuscript preparation, all analyses were performed using the original dataset with accurately assigned diabetes phenotypes, and these corrections did not compromise the validity of the results. Additionally, the careful selection of T2DM recipients may introduce selection bias and limit the applicability of our findings to broader, less selected T2DM populations. Furthermore, the relatively short follow-up period restricts our conclusions to early postoperative outcomes and highlights the need for longer prospective studies to assess sustained metabolic differences and graft function. Given these limitations, particularly the sample size and its single-center nature, multicenter, prospective validation is needed to confirm these findings and to further elucidate subtle metabolic distinctions that may not be apparent in the current study. 

## Conclusions

In this retrospective, single-center study of carefully selected simultaneous pancreas-kidney transplantation recipients, early postoperative glycemic trajectories and short-term graft outcomes were equivalent between patients with type 1 diabetes mellitus (T1DM) and those with type 2 diabetes mellitus (T2DM). These findings support the metabolic safety and clinical feasibility of SPKT in appropriately selected T2DM patients and reinforce that early metabolic outcomes are comparable across diabetes phenotypes when standardized perioperative care is applied. Early postoperative metabolic assessment, including vigilant glucose monitoring at predefined time points, may serve as a meaningful indicator of early graft function and a guide for timely clinical decision-making.

While modest differences in renal function were observed at one month, the clinical relevance of this remains uncertain and warrants further investigation. Taken together, our results support the continued, thoughtful expansion of SPKT eligibility to include select T2DM recipients and underscore the importance of early postoperative metabolic evaluation to optimize patient-centered outcomes.
